# The enigma of DNA methylation in the mammalian oocyte

**DOI:** 10.12688/f1000research.21513.1

**Published:** 2020-02-25

**Authors:** Hannah Demond, Gavin Kelsey

**Affiliations:** 1 Epigenetics Programme, The Babraham Institute, Cambridge, UK; 2 Centre for Trophoblast Research, University of Cambridge, Cambridge, UK

**Keywords:** DNA methylation, oocyte, chromatin

## Abstract

The mammalian genome experiences profound setting and resetting of epigenetic patterns during the life-course. This is understood best for DNA methylation: the specification of germ cells, gametogenesis, and early embryo development are characterised by phases of widespread erasure and rewriting of methylation. While mitigating against intergenerational transmission of epigenetic information, these processes must also ensure correct genomic imprinting that depends on faithful and long-term memory of gamete-derived methylation states in the next generation. This underscores the importance of understanding the mechanisms of methylation programming in the germline. *De novo* methylation in the oocyte is of particular interest because of its intimate association with transcription, which results in a bimodal methylome unique amongst mammalian cells. Moreover, this methylation landscape is entirely set up in a non-dividing cell, making the oocyte a fascinating model system in which to explore mechanistic determinants of methylation. Here, we summarise current knowledge on the oocyte DNA methylome and how it is established, focussing on recent insights from knockout models in the mouse that explore the interplay between methylation and chromatin states. We also highlight some remaining paradoxes and enigmas, in particular the involvement of non-nuclear factors for correct *de novo* methylation.

## Mammalian oocytes have a distinctive epigenome

**Figure 1.**
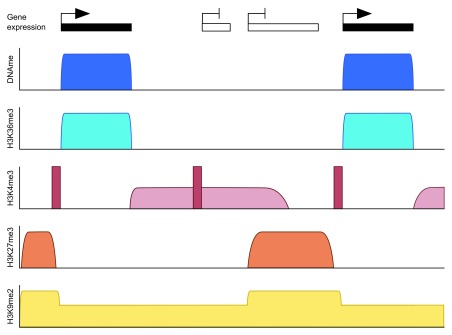
Schematic depicting the typical DNA methylation pattern of fully grown oocytes over active gene bodies and distribution of associated chromatin marks. While H3K36me3 is enriched over methylated domains, H3K27me3 and H3K9me2 are enriched in unmethylated domains. H3K4me3 has an unusual pattern in the oocyte in that peaks can be found at both active and inactive gene promoters (dark red) as well as enrichment over broad domains in untranscribed regions (light red). H3K27me3 is also broadly distributed over non-transcribed regions in oocytes but generally mutually exclusive to H3K4me3.

DNA methylation is considered a repressive epigenetic modification. In most mammalian somatic cells, it occurs across the majority of the genome, with the notable exception of active gene regulatory elements, such as promoters, enhancers, and CpG islands that are generally unmethylated; methylation of such elements represses their activity ^1^. Thus, DNA methylation is thought to function in the regulation of gene expression and in genome stability of heterochromatic regions. The oocyte is different in that both the genomic methylation pattern and its function are distinct from somatic cells. In oocytes, methylation is largely restricted to actively transcribed regions, including gene bodies, giving the oocyte genome a bimodal pattern of highly methylated gene bodies separated by intergenic or transcriptionally inactive regions with low levels of methylation ^2– 4^ ( Figure 1). This simplified pattern might be a consequence of the fact that only a single *de novo* methyltransferase—DNA methyltransferase (DNMT) 3A—is active in oocytes ^5^, but it also reflects that this is a newly acquired landscape, most pre-existing methylation having been erased. During mammalian embryo development, DNA methylation is globally erased in the primordial germ cells, which arise from cells of the epiblast. Thus, primary oocytes when they are first specified (which we can define as when a primordial germ cell enters and then arrests in the first meiotic prophase, in which state it remains until ovulation) are almost devoid of methylation ^2, 5– 7^. DNA methylation is re-set during the later stages of oocyte growth, culminating in the oocyte-specific pattern. The oocyte therefore represents a fascinating model system to study DNA methylation mechanisms because an entire methylation landscape is established from scratch in a non-dividing cell. As much of our knowledge of the mechanisms and function of oocyte methylation has come from mouse models, we will focus mainly on the mouse in this review. For a comprehensive comparison between human and mouse oocyte methylation, see 8.

The general restriction of methylation to actively transcribed gene bodies in the oocyte may indicate a distinct function. In somatic cells, gene-body methylation has been suggested to be one mechanism by which cryptic, intragenic promoters are repressed ^9^, although this conclusion has been questioned ^10^. But methylation probably has little influence in controlling gene activity in the oocyte itself because primary oocytes appear to initiate a faithful transcription programme before DNA methylation is put in place ^11^, and RNA-seq analysis of *Dnmt3L*-knockout oocytes that are effectively devoid of methylation indicate that there are no transcriptional differences compared with control oocytes ^2^. It might be that more careful analysis with improved RNA-seq methods could reveal some subtle effects. It is not evident, either, that methylation is required for repression of transposable element activity in oocytes. Where methylation in oocytes is absolutely essential for conferring DNA methylation is at imprinted genes. Imprinting is a process in which parent-of-origin allele-specific DNA methylation results in monoallelic expression of imprinted genes in offspring tissues ^12^. Most imprinted genes in the mouse and human have their imprinting control regions (ICRs or germline differentially methylated regions [DMRs]) methylated in oocytes. These ICRs are CpG islands that are intragenic in oocytes and become methylated during oocyte growth ^4^. Failure to establish methylation at ICRs in the oocyte results in severe developmental abnormalities ^13^, showing the importance of correct epigenetic programming in the oocyte for the next generation.

It might seem extravagant to methylate a large fraction of the genome to ensure methylation of a couple of dozen imprinted loci. However, *Dnmt3L*- and *Dnmt3a*-null oocytes, which lack most DNA methylation, are ostensibly normal, can be fertilised, and will support development of the embryo until beyond implantation, when imprinting defects cause developmental arrest ^13, 14^). But it is possible that methylation in the oocyte has significance beyond classical imprinted genes. Embryos developing from DNA methylation-deficient oocytes succumb to problems in trophoblast development, hence placental defects, for which a contribution of genes outside known imprinted genes has been implicated ^15^. Thus, it seems likely that there are more extensive effects of oocyte methylation than classical imprinted genes.

## *De novo* methylation requires active transcription

Consistent with the predominant gene-body pattern of methylation, *de novo* DNA methylation in the oocyte has been shown to require active transcription. Knockout experiments in the mouse have shown that abrogating transcription across individual genes results in failure to establish methylation at these loci ^4, 16– 18^. These examples focus on imprinted genes, whose imprinting status depends on the acquisition of DNA methylation at their ICRs. Similar conclusions have been drawn from human studies, in which chromosome deletions or rearrangements that are likely to result in interrupting transcription across imprinted domains lead to imprinting disorders ^19– 21^. This sets up the important premise that whether or not a gene is transcribed in oocytes can determine how it is regulated in the next generation.

Because of the predominant connection with transcription, species-specific variations in the oocyte transcriptome become reflected in variations in the oocyte methylome. A significant proportion of transcription units in oocytes have been found to be determined by alternative promoters ^4^. Strikingly, more than a third of oocyte-specific transcription units initiate from active long-terminal repeats (LTRs) of endogenous retroviral elements, notably of the MaLR and endogenous retrovirus K (ERVK) classes ^4, 22, 23^. As LTR insertions differ between species, and even between strains of mice, the ensuing oocyte methylation patterns differ ^23^. This variation can also provide the basis for differences in imprinted genes between human and mouse ^24^.

## Mechanistic predictions of DNMT targeting

There appears to be a simple logic to the methylation pattern that arises in oocytes, which is informed by the biochemical properties of the *de novo* methyltransferases involved: in mouse oocytes, this is DNMT3A as the active enzyme and the related DNMT3L as an essential auxiliary factor. Ablation of either in oocytes abolishes *de novo* methylation to similar degrees ^2, 5, 6^. Both proteins have limited DNA binding specificity; instead their binding and activity is modulated by the post-translation modification state of histone proteins. Notably, both DNMT3A and DNMT3L bind the amino-terminal domain of histone H3 through their plant homeodomain (PHD)/ADD domains, but not when lysine 4 (H3K4) is methylated ^25^. Recognition of unmethylated H3K4 releases DNMT3A from an autoinhibitory conformation ^26^. In addition, the Pro-Trp-Trp-Pro motif (PWWP) domain of DNMT3A (there is no equivalent domain in DNMT3L) binds H3K36me2/3, at least *in vitro* ^27, 28^. DNMT3A may also interact with H3K27me3 ^29, 30^, but this interaction does not apply to the isoform expressed in oocytes.

The antagonistic and permissive interactions of DNMT3A/DNMT3L with H3K4me3 and H3K36me3, respectively, set up a simple scenario that could account for the gene-body methylation pattern characteristic of oocytes. H3K36me3 should be enriched over active gene bodies; H3K4me3 should demarcate active promoters and other CpG islands ^31^. Intergenic or non-transcribing regions should have neither mark, such that these regions are neutral to the DNMT3A/DNMT3L complex. Chromatin immunoprecipitation and sequencing (ChIP-seq) profiles for mouse (and human) oocytes that have emerged in recent years can be interpreted largely to support this model: DNA methylation domains coincide with regions of H3K36me3 enrichment, and DNA methylation is absent from or much reduced in sites of H3K4me3 enrichment ^32– 35^ ( Figure 1). Intriguingly, ChIP-seq data have revealed unusual properties of chromatin in mouse oocytes, in particular a widespread accumulation of H3K4me3 over intergenic and low methylated domains ^32– 34^.

## What have mouse knockout models told us?

**Figure 2.**
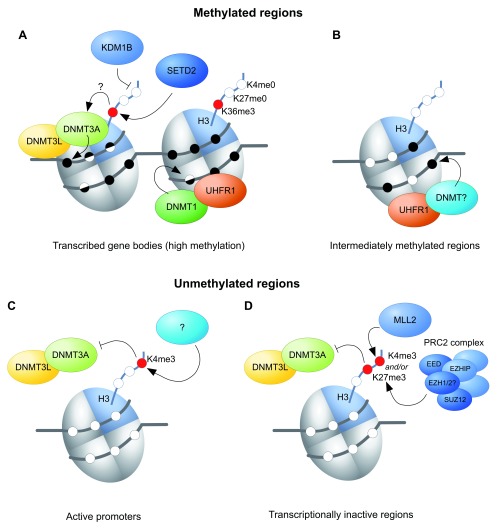
Models showing chromatin factors involved in DNA methyltransferase (DNMT) 3A/DNMT3L recruitment in methylated regions and factors inhibiting DNMT3A/DNMT3L binding at unmethylated regions. **A**) At actively transcribed gene bodies, SETD2-mediated H3K36me3 has been proposed to recruit DNMT3A/DNMT3L, whilst lysine demethylase 1B (KDM1B) seems to be required to prevent or remove histone 3 lysine 4 (H3K4) methylation. DNMT1 is needed for methylation of hemimethylated DNA. **B**) Ubiquitin-like, plant homeodomain and ring finger-containing 1 (UHRF1) is required for intermediate DNA methylation of some genic and intergenic regions, likely by recruiting one of the DNMT proteins, but the chromatin requirements are unknown. **C**) At active promoters, H3K4me3 is thought to prevent DNMT3A/DNMT3L binding. **D**) Transcriptionally inactive regions can be marked by mixed lineage leukaemia-2 protein (MLL2)-mediated H3K4me3 and/or polycomb repressive complex 2 (PRC2)-mediated H3K27me3, preventing recruitment of DNMT3A/DNMT3L to these regions. EED, embryonic ectoderm development; EZHIP, EZH inhibitory protein; SETD2, SET domain containing 2.

More definitive tests of the model above have come from gene knockouts in the mouse. H3K36me3 is conferred by a single, conserved enzyme, SETD2, which associates with the elongating RNA polymerase II, thus depositing H3K36me3 over transcribed gene bodies and recruiting the DNA methylation machinery ( Figure 2A). CpG islands are normally depleted in H3K36me3, but during the course of oocyte growth, intragenic CpG islands destined for DNA methylation gain H3K36me3 enrichment ^36^. Oocyte-specific ablation of SETD2 causes loss of H3K36me3 and, as expected, gene-body methylation is eliminated, as too is methylation of ICRs ^35^. Rather surprisingly, however, there is appearance of DNA methylation in regions of the genome ordinarily unmethylated, as if DNMT3A/DNMT3L becomes more promiscuous in the absence of its favoured mark. It is possible that this off-target activity reflects the opportunity presented by the length of time over which *de novo* methylation can occur in oocytes, which may be several days during the latter stages of follicular development and oocyte growth.

Evidence for a role for active removal of H3K4 methylation first came with the knockout of lysine demethylase 1B (KDM1B) (AOF1), one of the two known H3K4me2 demethylases ^37^. Single-locus analysis indicated that some imprinted gDMRs failed to become methylated in *Kdm1b*-null oocytes ^37^. Subsequent genome-wide interrogation of methylation showed that most imprinted ICRs exhibit reduced methylation in *Kdm1b* knockout oocytes, as do many CpG islands that normally become methylated in oocytes ^36^ ( Figure 2A). Why some imprinted ICRs are more sensitive to the loss of KDM1B is not fully known, but there is some evidence that ICRs and CpG islands acquiring methylation later in oocyte growth are more susceptible ^11, 36, 37^.

So far, there is no indication of a specific involvement of an H3K4me3 demethylase for *de novo* DNA methylation, despite the expectation that CpG islands destined for DNA methylation are initially enriched in H3K4me3 in primary oocytes ^36^. Of the H3K4me3 demethylases, lysine demethylase 5C (KDM5C) (JARID1C) is the most abundantly expressed at the transcriptional level in mouse oocytes, but oocyte-specific ablation of KDM5C does not impair *de novo* DNA methylation (Huang and Kelsey, unpublished data). It is possible that the necessary removal of H3K4me3 from CpG islands could be accomplished by nucleosome replacement as part of the transcription process. Ablation of the chaperone protein HIRA responsible for incorporation of H3/H4 outside of DNA replication dramatically reduces DNA methylation ^38^, which could attest to the contribution of nucleosome remodelling in *de novo* DNA methylation.

As noted above, oocyte chromatin is unusual in that H3K4me3, which is normally highly localised to active promoters, accumulates over intergenic/non-transcribed domains during oocyte growth ^32– 34^ ( Figure 1). Accordingly, there could be an expectation that this pervasive H3K4me3 would constrain deposition of DNA methylation ^32^, noting that H3K4me3 and DNA methylation are accumulating in the oocyte genome with similar kinetics ^34^. However, ablation of mixed lineage leukaemia-2 protein (MLL2/lysine methyltransferase 2B [KMT2B]), which is responsible for the bulk of intergenic H3K4me3, results in very limited ectopic DNA methylation ^34^. This result would tend to argue that absence of the antagonistic H3K4me3 modification is insufficient to promote genomic binding of DNMT3A/DNMT3L and recruitment depends more on an attractive mark, such as H3K36me3. In contrast, removal of DNA methylation from oocytes through ablation of DNMT3A results in further spreading of H3K4me3 into ordinarily methylated domains, suggesting that genomic recruitment of MLL2 is predominantly determined by the availability of unmethylated CpG sites ^34^.

Canonical H3K4me3 is present at active promoters in the oocyte and thought to prevent DNA methylation at those sites ( Figure 2C). Canonical H3K4me3 does not depend on MLL2, and the methyltransferase catalysing this mark is still unknown. Besides H3K4me3, H3K27me3 may also prevent DNMT3A/DNMT3L from methylating DNA, as H3K27me3 and DNA methylation are generally found to be mutually exclusive, including in oocytes ( Figure 2D) ^39^. Although mouse knockouts of members of the polycomb repressive complex 2 (PRC2), such as embryonic ectoderm development (EED) and the germ-cell-specific EZH inhibitory protein (EZHIP), have been described, so far they have not been evaluated for effects on DNA methylation in the oocyte ^40– 42^.

The repressive modifications H3K9me2 and H3K9me3 have often been associated with DNA methylation. In the oocyte, H3K9me2 is enriched in regions devoid of DNA methylation ^43^. In line with this, ablating one of the major euchromatin H3K9me2 methyltransferases, G9A/euchromatin histone methyltransferase 2 (EHMT2), has very limited impact on DNA methylation in oocytes ^43^. Similar to other cell types, H3K9me3 in the oocyte is associated with DNA methylation and both marks can be found at silenced developmental genes ^44^. However, there is no current evidence suggesting that *de novo* methylation depends on H3K9me3. Instead, both H3K9me2 and H3K9me3 have been proposed to be involved in DNA methylation maintenance, as the DNMT1 accessory protein ubiquitin-like, PHD and ring finger-containing 1 (UHRF1) has a binding domain for H3K9me3 ^45– 48^.

The precise timing and dependencies of the chromatin events required to ensure faithful DNA methylation are still not fully understood. For example, is a specific activity required to enable H3K36me3 to accumulate at intragenic CpG islands, or is it accomplished by nucleosome turnover/replacement associated with transcription? The interdependence of many of the processes makes testing all elements of the model difficult. For example, earlier work in which transcription across the imprinted *Zac1* locus was ablated indicated that transcription was required for not only deposition of H3K36me3 but also removal of H3K4me2 at the ICR-CpG island ^4^. This could be interpreted as a requirement for transcription to recruit the H3K4me2 demethylase KDM1B, and, indeed, full methylation at this ICR does depend on KDM1B ^36, 37^. But, from the *Setd2* knockout ^35^, it is also possible that ablation of H3K36me3 from the region leads to ectopic marking of H3K4me3 or H3K27me3 across the locus, which would be antagonistic to DNA methylation.

## Methylation independent of transcription

**Figure 3.**
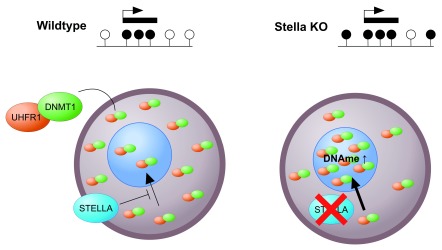
The role of STELLA in regulating methylation in the oocyte. In the oocyte, STELLA is required for the cytoplasmic localisation of the majority of DNA methyltransferase 1 (DNMT1)/ubiquitin-like, plant homeodomain and ring finger-containing 1 (UHRF1). Ablation of STELLA results in UHRF1/DNMT1 redistribution into the nucleus, resulting in increased DNA methylation at regions that are normally unmethylated. KO, knockout.

We have focussed above on mechanisms that explain hypermethylated domains over gene bodies; however, a small fraction of the oocyte methylome falls outside of transcribed regions. Methylation of these regions depends on the DNMT1 accessory protein UHRF1 ^49^, but the mechanism is not understood ( Figure 2B). Correlation with chromatin maps may now help to suggest mechanistic links. The above also portrays unmethylated regions as being completely passive and remaining unmethylated because there is nothing to recruit DNMT3A/DNMT3L to these regions (under normal circumstances). Intriguingly, ablation of the protein STELLA (DPPA3, primordial germ cell 7 [PGC7]) leads to pronounced aberrant gain of methylation of intergenic, unmethylated domains ^50^, and this depends on UHRF1 and DNMT1. This finding leads to the model in which STELLA is required to prevent nuclear activity of DNMT1/UHRF1 in oocytes ( Figure 3). Nevertheless, DNMT1 does seem to be required to fill in the gaps in methylation left by DNMT3A ^5^, i.e. to ensure symmetric methylation of CpG sites, thus demonstrating a role for DNMT1 outside of DNA replication ( Figure 2A).

## DNMT3 paradoxes

The model above indicates a role for H3K36me3 in recruiting DNMT3A to active gene bodies, which was predicted to be mediated via the PWWP domain of DNMT3A ^27^. So far, this prediction has not been confirmed. Genomic studies in mouse embryonic stem cells (ESCs) have not identified binding of DNMT3A at H3K36me3-marked domains ^51^. Moreover, a point-mutation in the DNMT3A-PWWP domain designed to abrogate the *in vitro*-defined interaction with H3K36me3 ^27, 52^ does not cause reduced gene-body methylation in oocytes (Sendzikaite and Kelsey, unpublished data). Instead, DNMT3B has been shown to bind and functionally methylate H3K36me3 domains in ESCs ^51^, but DNMT3B is functionally insignificant in mouse oocytes ^5^. Recently, it was shown that DNMT3A is recruited to sites of H3K36me2 in C3H10T1/2 mesenchymal stem cells (MSCs) ^53^. However, the genomic distribution of H3K36me2 in oocytes has not been reported, and it is unclear whether it should be restricted to gene bodies, as it was found to have a more diffuse distribution than H3K36me3, encompassing both genic and intergenic regions in MSCs.

Another paradox concerns the role of DNMT3L. In the mouse, ablation of DNMT3L results in failure of *de novo* methylation in oocytes ^5, 6, 13^. DNMT3L has no catalytic activity, so its requirement indicates an essential role in forming heterotetramers with DNMT3A ^54^. However, RNA-seq analysis shows that DNMT3L is not transcribed in human oocytes ^3^. Instead, it is possible that DNMT3B substitutes as a partner for DNMT3A. Intriguingly, it has recently been shown that although DNMT3B is essential for a major portion of the *de novo* methylation occurring in early post-implantation mouse embryos, this role can largely be provided by a catalytically inactive mutant of DNMT3B ^55^. This study supports previous findings from somatic cell lines, also suggesting that catalytically inactive isoforms of DNMT3B can fulfil a structural role as an accessory protein in helping to recruit other DNMTs, similar to DNMT3L ^56^. Therefore, it might be that shorter, non-catalytic isoforms of DNMT3B replace DNMT3L in human oocytes as a partner for DNMT3A.

## Factors yet to factor in

In addition to the factors highlighted above—transcription, chromatin, DNA methyltransferases—other molecular or cellular components will be necessary for correctly establishing the DNA methylation pattern distinctive in oocytes. It might be anticipated that factors required to provide the metabolites for *de novo* methylation—S-adenosylmethionine (SAM) as the universal donor for the methylation reaction, but also other intermediary metabolites such as α-ketoglutarate required as a co-factor for lysine demethylases—could be rate-limiting in certain circumstances, such as nutritional deprivation. At this time, there is no real evidence that this is the case, but changes in cellular metabolism have been linked to DNA methylation changes in other contexts. For instance, in somatic cells, mitochondrial depletion has been shown to alter methionine metabolism, resulting in increased SAM levels and consequent increase in DNA methylation ^57^. It remains to be seen whether less dramatic, but more pervasive, nutritional imbalances (and their physiological consequences), such as maternal high-fat diet, alter oocyte methylation in a manner that can be rationalised by a biochemical mechanism. The availability of quantitative and sensitive methylation profiling methods, including at the single-cell level ^58^, should now enable these questions to be asked at a genome-wide level without preconceptions about the types of sequences that could be affected. Moreover, the knowledge of the underlying mechanism of *de novo* methylation and its relationship to chromatin properties should provide a rational explanation for any effects observed. Finally, the detailed knowledge we also have of how DNA methylation is reprogrammed during preimplantation embryo development should enable us to infer to what extent DNA methylation effects observed in oocytes could influence developmental progression or gene expression in the embryo.

A factor that does have an essential role in DNA methylation, but by still-obscure mechanisms, is the subcortical maternal complex (SCMC). This is a multi-protein complex, whose constituents are abundantly expressed during oocyte growth and which becomes elaborated as a distinct structure under the oocyte plasma membrane ^59, 60^. It retains this configuration in blastomeres of cleavage embryos. The SCMC is essential for a number of vital processes during the egg-to-embryo transition, such as cellular organisation in the oocyte and mitotic cleavages in the early embryo ^60^. Intriguingly, in humans, it also has an impact on the fidelity of DNA methylation in the oocyte and/or preimplantation embryo. This has been indicated by maternal-effect mutations in several components of the SCMC that are associated with imprinting defects or gestational trophoblast diseases ^61– 67^. Until recently, it was not known whether mutations in these proteins impaired *de novo* methylation in the oocyte or interfered with DNA methylation maintenance in the cleavage embryo at a time of genome-wide DNA methylation programming during which methylation at imprints has to be maintained. Very recently, using single-cell bisulphite sequencing, we have shown that oocytes with a mutation in the SCMC protein KH domain containing 3-like (KHDC3L) have severely impaired methylation throughout the genome ^68^. Although this is an important advance in knowledge because it identifies the stage at which methylation defects originate, it will be very challenging to elucidate the mechanism by which SCMC defects compromise DNA methylation establishment, given the lack of cellular models to explore these mechanisms in the oocyte. It is unclear whether the murine SCMC is also involved in DNA methylation establishment or imprint maintenance. Mouse knockout models of most of the SCMC proteins result in very early embryo arrest, and analysis has not focused on DNA methylation ^59, 69, 70^. Loss of NLR family pyrin domain containing 2 (NLRP2) was shown to alter the localisation of DNMT1 but not DNMT3A in the oocyte, but only minor and inconsistent changes in the methylation of imprinted genes were observed in embryos or newborn offspring ^71^. A closer assessment of mouse SCMC proteins and their role in DNA methylation will be required to determine whether the SCMC has a similar function in mice as in humans and whether mouse knockout models could help to elucidate the mechanism by which it regulates DNA methylation in the oocyte.

## Conclusions

The mammalian oocyte has a deceptively simple DNA methylation landscape that is likely to reflect transcription and associated chromatin remodelling events. Whole-genome sequencing studies assessing DNA methylation, transcription, and histone modifications in combination with gene knockouts in the mouse have supported early hypotheses, but atypical distribution of some histone modifications, as well as unexpected findings in the knockout models, mean that the involvement of some factors remains unclear. In addition to elucidating the factors involved in DNA methylation in the mouse, future studies will also have to link the effect of the unusual transcriptional and epigenetic landscape of the oocyte to the embryo. Furthermore, it has become clear that although the link between transcription and DNA methylation seems to be upheld in human oocytes, other factors, such as the DNMTs and SCMC members, may be differentially regulated in humans.

## Abbreviations

ChIP-seq, chromatin immunoprecipitation and sequencing; DMR, germline differentially methylated region; DNMT, DNA methyltransferase; ERVK, endogenous retrovirus K; ESC, embryonic stem cell; ICR, imprinting control region; H3K4, histone 3 lysine 4; KDM1B/ *Kdm1b*, lysine demethylase 1B; KDM5C, lysine demethylase 5C; LTR: long-terminal repeat; MLL2, mixed lineage leukaemia-2 protein; MSC, mesenchymal stem cell; PHD, plant homeodomain; PRC2, polycomb repressive complex 2; PWWP, Pro-Trp-Trp-Pro motif; SAM, S-adenosylmethionine; SCMC, subcortical maternal complex; UHRF1, ubiquitin-like, PHD and ring finger-containing 1
